# P-glycoprotein efflux transporter: a key to pharmacokinetic modeling for methadone clearance in fetuses

**DOI:** 10.3389/fphar.2023.1182571

**Published:** 2023-05-04

**Authors:** Harvey Ho, En Zhang

**Affiliations:** ^1^ Auckland Bioengineering Institute, The University of Auckland, Auckland, New Zealand; ^2^ Chongqing Food and Drug Control Institute, Chongqing, China

**Keywords:** methadone, opioids, PBPK, P-glycoprotein, model

## Background

The situation of opioid abuse has deteriorated in recent years. In 2019, 49,860 people died from opioid overdoses in the USA alone, a 6-fold increase since 2000 (Ko et al., 2020). Of particular concern is opioid abuse (e.g., morphine, fentanyl) by pregnant women. It was found that 6.6% of women self-reported opioid use during pregnancy in the USA, and 21.2% of them disclosed opioid misuse (Ko et al., 2020). Infants born to drug-abusing mothers are at risk of preterm delivery, poor intrauterine growth, and neonatal abstinence syndrome (NAS). Currently, Methadone Maintenance Treatment (MMT) is the recommended therapy for opioid addicts, including pregnant women (World Health Organization, 2014). However, the pharmacokinetics of methadone is greatly affected by pregnancy. Physiological changes during pregnancy (e.g., increases in total body fluid, blood volume, and body fat) and metabolic enzyme activities (CYP2B6, CYP3A4) lead to faster clearances of methadone in pregnant women (Badhan and Gittins, 2021). Consequently, a higher dosage may be required during pregnancy. Some researchers suggest a staged escalation approach, i.e., starting from the lowest pre-natal dose (30 mg once daily) to the maximum 120, 140, and 180 mg daily dose for trimester 1, 2 and 3, respectively (Badhan and Gittins, 2021). This is significantly higher than a standard dosage (15–60 mg/day) and may expose fetuses and neonates to higher risks of NAS. Indeed, of all infants born to mothers receiving MMT, 40%–90% show signs of NAS. For example, in a retrospective study of 67 women (40 received MMT at 10–20 mg/day), it was found that 43% of the infants of mothers receiving MMT required treatment for NAS (Malpas et al., 1995). Similar results were also reported in (Dryden et al., 2009). However, it was also found that there was no strong correlation between the dosage of methadone use and the severity of NAS (Malpas et al., 1995; Berghella et al., 2003).

**FIGURE 1:**
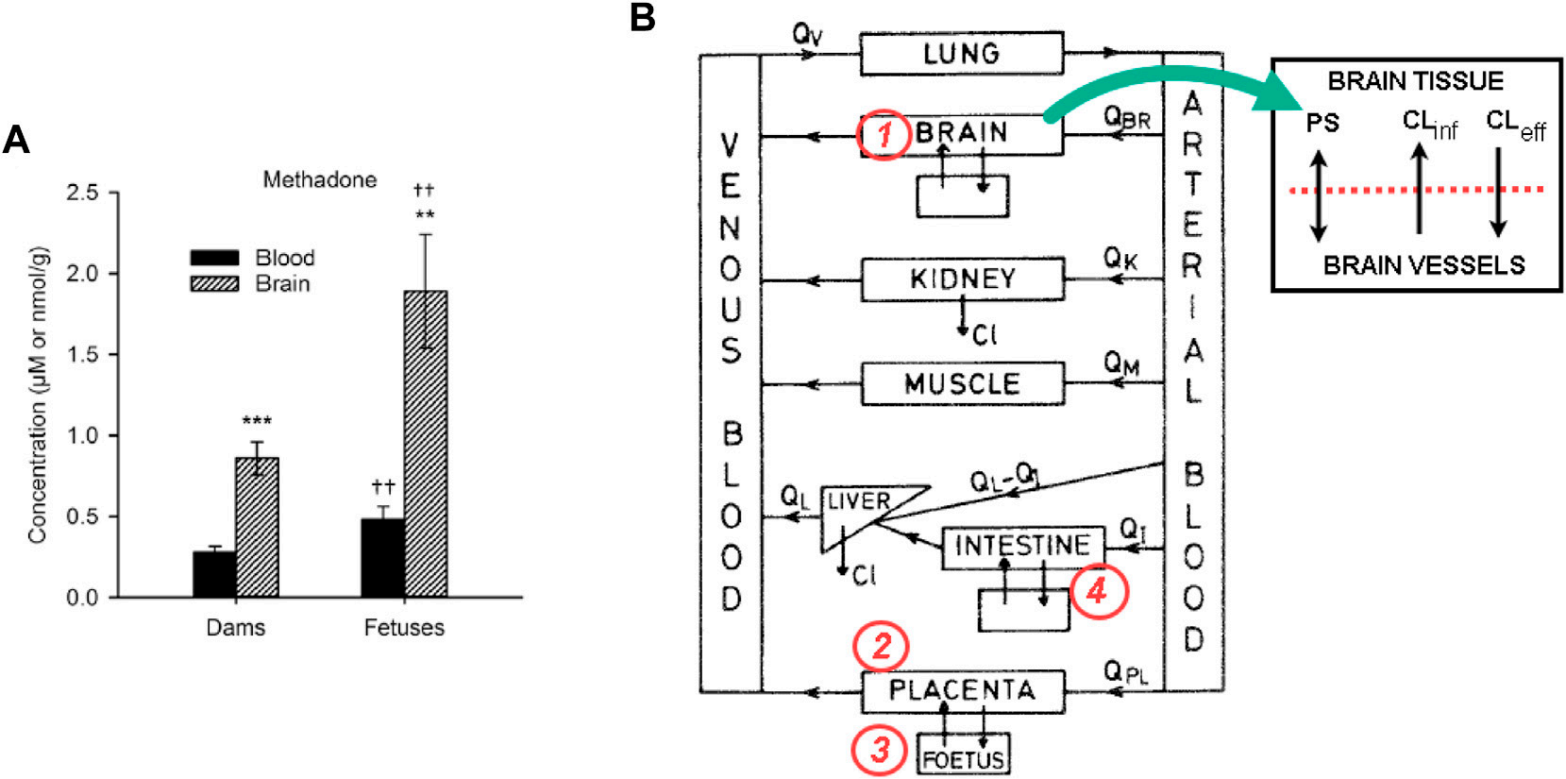
**(A)** The methadone concentrations in the blood and brain of fetuses were 1.6 and 2.8 times higher than in dams, respectively. Figure adopted from Kongstorp et al. (2019); **(B)** The diagram for a f-m PBPK model for methadone, and the proposed changes to incorporate the P-gp inhibition/induction kinetics. Equations for at least four compartments (the brain, intestine, placenta, and fetus) of the model need to be revised.

In a recent pregnant rat model, it was shown that the methadone concentrations in the blood and brain of fetuses were 1.6 and 2.8 times higher than in dams, respectively (Kongstorp et al., 2019) (Figure 1A). The finding highlights the susceptibility of fetuses to maternal methadone exposure. Being lipophilic and with a low molecular weight (309 Da), methadone readily crosses the placenta barrier via passive diffusion. However, the return of methadone from the fetal side to the maternal side (f→m) also requires active transport by the efflux transporter P-glycoprotein (P-gp) (Nanovskaya et al., 2005). P-gp is expressed at the apical membrane of cotyledons, i.e., the maternal side (Gil et al., 2005; Nanovskaya et al., 2005). Therefore, the placenta acts as barrier that retards the backflow of methadone f→m, which causes a higher plasma concentration of methadone at the fetal side than the maternal side (Figure 1A). To assist the clearance of methadone from the fetal side, P-gp acts as the efflux transporter (f→m), on top of the limited passive perfusion. Therefore, an inhibition of P-gp would deteriorate the accumulation of methadone at the fetal side. The phenomenon has been demonstrated in experiments. For example, in an *ex vivo* placental perfusion experiment, it was shown that inhibition of P-gp led to a 30% increase in methadone in fetal circulation (Nanovskaya et al., 2008). Similar to its role as an efflux transporter in the placenta, P-gp is expressed at the blood-brain barrier (BBB) and acts as an efflux transporter for xenobiotics from brain tissues back to the cerebral flow. Previous studies of P-gp mediated methadone perfusion across the BBB are rarely reported. However, inhibition of P-gp greatly increased the toxicity of another synthetic opioid, fentanyl, in a rat brain model (Yu et al., 2018). The aggregated efflux effects of P-gp on the placental and BBB barriers lead to the higher concentrations in fetal brain not only higher that the maternal brain tissues, but also higher than fetal circulations in rat models (Figure 1A) (Kongstorp et al., 2019).

Based on these observations, it is hypothesized that by inducing P-gp at the placental barrier and BBB, the methadone concentration in the fetal blood and brain can be reduced. Consequently, symptoms of CNS may be alleviated. However, co-administration of a P-gp inducer (e.g., Rifamacin) with methadone also induces metabolic enzymes, e.g., CYP3A4, which can lead to a more rapid clearance of methadone and possible withdrawal syndromes in mothers. In addition, a P-gp inhibitor or inducer acting on the intestine barrier may change the bioavailability of methadone if it is administered orally (Carlos et al., 2002). This is a typical drug-drug interaction (DDI) scenario between methadone, P-gp and cytochrome P450 (CYP) enzymes in a specific population (pregnant women and fetuses), where there is a significant knowledge gap.

In this opinion paper, we first provide a brief review of some *in vivo*, *in vitro*, and *ex vivo* models for the role P-gp plays in methadone clearance in fetuses. We then highlight P-gp as a key in fetal-maternal physiologically based pharmacokinetic (PBPK) and *ex vivo* placenta perfusion models, and propose computational methods for incorporating efflux transporter kinetics in pharmacokinetic models.

### *In vivo* animal models

As a synthetic opioid agonist, methadone is mainly eliminated via hepatic metabolism by CYP 2B6, and partly by CYP 3A4 (Garrido and Trocóniz, 1999; Kharasch, 2017). Its concentration in the blood has a long half-life (20–35 h), which is crucial to prevent withdrawal symptoms for at least 24 h. *In vivo* models for fetal exposure to methadone are mainly performed in animals in particular on rats (Gabrielsson et al., 1985; Chen et al., 2015; Kongstorp et al., 2019; Kongstorp et al., 2020). In these models, administering methadone is either via subcutaneous injection (Chen et al., 2015), or through an implanted osmotic minipump to keep constant opioid exposure in dams (Kongstorp et al., 2019). The dose regimen was 10 mg/kg/day, or 1.0 mL/kg of body weight to induce stable blood concentrations of 0.25 ± 0.02 μM methadone in the pregnant rats, which are comparable to the concentrations reported in pregnant women in MMT. However, *in vivo* data on P-gp mediated fetal exposure to methadone are rare. For example, in a literature review of fetal and offspring exposure to opioids in animals and humans, there was no mention of either inhibition or inducing P-gp (Farid et al., 2008).

### *In vitro* cell line models

Monolayer BeWo cell lines have been used to investigate the permeability of opioids in the placental trophoblast (Nanovskaya et al., 2005; Mortensen et al., 2019). A study investigated the BeWo cell permeability of six opioids (Mortensen et al., 2019), and it was found that the permeability of methadone is lower than oxycodone but higher than morphine and heroin. Another study revealed methadone uptake was increased in the presence of the P-gp inhibitor cyclosporin A (Nanovskaya et al., 2005), reflecting a reduced methadone efflux. In addition, it has been shown that the basolateral to apical transport of known p-glycoprotein substrates (vinblastine, vincristine, and digoxin) is significantly greater than transport to the opposite direction (Ushigome et al., 2000). This is consistent with what has been found in the *ex vivo* placenta in perfusion experiments (Nanovskaya et al., 2008). Although it is rare, human intestinal cell line Caco-2 was used to investigate transporter-mediated opioid transfer in BBB. The efflux ratio of fentanyl was remarkably reduced when co-incubated with tariquidar, a P-gp inhibitor (Yu et al., 2018). However, the prediction of drug transport through the BBB *in vivo* may not be accurate since the BBB, and the intestinal musosa are two fundamentally different biologic barriers (Lundquist et al., 2002). Instead, *in vitro* models of BBB with monolayer of brain capillary endothelial cells were shown to have a good correlation with *in vivo* rat models (Lundquist et al., 2002).

### *Ex vivo* placental models

*Ex vivo* placental perfusion is considered the “golden standard” for examining the transplacental properties of an investigational drug. In the experiment setup, a single unit of cotyledon is cannulated and connected to perfusates at the maternal and fetal sides separately (Nanovskaya et al., 2008) (Malek et al., 2009) (Kurosawa et al., 2020). In one such study (Nanovskaya et al., 2008), methadone concentrations were measured from the perfusate reservoirs and at sampling ports. It was found that in the maternal to fetal (m→f) perfusion route, there was a biphasic decrease in methadone concentration, i.e., a sharp drop in the first 60 min followed by a plateau in the remaining 180 min. In contrast, the f→m transperfusion showed a gradual decrease of methadone in the fetal artery. The opiate was retained by the placental tissue from both the m→f and the f→m routes.

It is worth pointing out that since placentas are usually collected postpartum, the *ex vivo* perfusion results only reflect the placental physiology at term, but not at the early stages of gestation. For example, the P-gp expression is lower at the apical membrane in trimester 3 than in other trimesters, which leads to a weaker efflux transport, and consequently a higher methadone accumulation in trimester 3 (Gil et al., 2005). This is unfortunate because the last stage of gestation is also the most crucial time for lowering fetal exposure to opioids and reducing the risk of NAS.

### *In silico* models

PBPK models are computational tools that quantify the time course of drug concentration in various organs and tissues, represented by compartments (Figure 1B). Fetal-maternal (f-m) PBPK models add compartments of the placenta and fetal organs into a standard model. f-m PBPK models have been developed for many drugs, including methadone (Gabrielsson et al., 1985; Ke et al., 2014). However, to our knowledge, the only pregnant rat model of methadone that has both *in vivo* experiments and f-m PBPK modelling was developed almost 40 years ago (Gabrielsson et al., 1985). Unfortunately, no DDI mechanism was investigated in that study.

In order to incorporate efflux transporter kinetics in the PBPK scheme shown in Figure 1B, four key compartments, namely, the brain, the placenta, the fetus and the intestine need to be revised (refer to Figure 1B). For the brain compartment, the transport-mediated DDI for BBB was implemented in two compartments, i.e., brain tissue and brain vasculature (Ball et al., 2012), where a passive diffusion term and two transport terms (representing active influx and efflux) are used to simulate the transport. A more complex four-compartment model was proposed in (Gaohua et al., 2016), where cranial and spinal cerebrospinal fluid compartments are added. The placenta compartment can be treated in a similar way, i.e., with apical and basolateral membranes in cotyledons, as has been used in an f-m PBPK model for nicotine (Amice et al., 2021). If methadone is administered orally, such as the liquid formulation being practiced in MMT clinics, then the intestine barrier needs to be implemented in the PBPK model. The pharmacokinetics of methadone in this case is different from when it is administered through infusion or intravenous injection (Chen et al., 2015). For instance, for the administration route of methadone, instead of intravenou*s* injection, an oral dosing regimen will also be used for comparison. It is worth noting that adding the fetal liver into the fetus compartment may not be significant for methadone from ontogeny’s perspective, since some of the major metabolic enzymes (CYP2B6, CYP3A4) are still not mature in fetuses, while other enzymes such as CYP3A7 is expressed in the fetal liver that can metabolise methadone (Wolff et al., 2005). Nevertheless, the fetal liver compartment is important for some other opioids including morphine and buprenorphine, because fetal liver enzymes, e.g., SULT1A3 and SULT2A1 that are involved in these opioids’ sulfation are already active.

A more thorough investigation of the transport properties of methadone in the placenta would require considering several membranes that separate the maternal and fetal circulations. To date, one of the most detailed mathematical models for *ex vivo* placenta perfusion is that developed for metformin (Kurosawa et al., 2020) and for morphine (Ho et al., 2022). The significance of the work is that the important data of P-gp mediated methadone transport were published (Nanovskaya et al., 2008), which allows an *in silico* model to be calibrated. Furthermore, the parameters in *in silico* models can be tuned per the reduced P-gp expressions as gestation progresses (Nanovskaya et al., 2005).

### Need for integrating *in silico*, *in vitro,* and *in vivo* models

In the above section, we have briefly discussed the current *in vivo*, *in vitro,* and *in silico* models for fetal exposure to methadone, in particular when considering the variable of efflux transporter P-gp. We also described a strategy for incorporating efflux kinetics into PBPK and *ex vivo* perfusion models. It is clear that none of these models is comprehensive in addressing the multiple facets of the methadone clearance mechanism in pregnant women and fetuses. Still, these models need to provide evidence-based methodology change for the current MMT practice, where clinicians rely on clinical symptoms (withdrawal or overmedication) when prescribing methadone. Efforts need to be made to fill the gaps and challenges. For example, extrapolation of *in vivo* placenta transfer from animal models to humans is challenging due to the anatomical and functional specificity of the placenta among different species (Myllynen and Vähäkangas, 2013). Similarly, *in vitro* cell line models may fall short of the prediction of *in vivo* drug transport (Lundquist et al., 2002). *Ex vivo* placenta perfusion models can only predict opioid transport properties at term, yet such properties are clearly altered by varying P-gp expression during pregnancy. While *in silico* models are cost-effective, they are prone to mathematical maneuver or parameter fitting when lacking data (Ho et al., 2022). Nevertheless, there is a more pressing need than ever to integrate the data and know-how gained from multiscale, multidisciplinary models in the era of the “opioid epidemic” and its treatment.
